# Spatial Dynamics of Bovine Tuberculosis in the Autonomous Community of Madrid, Spain (2010–2012)

**DOI:** 10.1371/journal.pone.0115632

**Published:** 2014-12-23

**Authors:** Maria Luisa de la Cruz, Andres Perez, Javier Bezos, Enrique Pages, Carmen Casal, Jesus Carpintero, Beatriz Romero, Lucas Dominguez, Christopher M. Barker, Rosa Diaz, Julio Alvarez

**Affiliations:** 1 Centro de Vigilancia Sanitaria Veterinaria VISAVET, Universidad Complutense, Madrid, Spain; 2 Department of Veterinary Population Medicine, University of Minnesota, St. Paul, Minnesota, United States of America; 3 MAEVA SERVET SL, Alameda del Valle, Madrid, Spain; 4 Área de Ganadería, Dirección General de Medio Ambiente, Consejería de Medio Ambiente, Vivienda y Ordenación del Territorio de la Comunidad de Madrid, Madrid, Spain; 5 Departamento de Sanidad Animal, Facultad de Veterinaria, Universidad Complutense, Madrid, Spain; 6 Center for Vectorborne Diseases and Department of Pathology, Microbiology, and Immunology, School of Veterinary Medicine, University of California Davis, Davis, California, United States of America; Institut de Génétique et Microbiologie, France

## Abstract

Progress in control of bovine tuberculosis (bTB) is often not uniform, usually due to the effect of one or more sometimes unknown epidemiological factors impairing the success of eradication programs. Use of spatial analysis can help to identify clusters of persistence of disease, leading to the identification of these factors thus allowing the implementation of targeted control measures, and may provide some insights of disease transmission, particularly when combined with molecular typing techniques. Here, the spatial dynamics of bTB in a high prevalence region of Spain were assessed during a three year period (2010–2012) using data from the eradication campaigns to detect clusters of positive bTB herds and of those infected with certain *Mycobacterium bovis* strains (characterized using spoligotyping and VNTR typing). In addition, the within-herd transmission coefficient (β) was estimated in infected herds and its spatial distribution and association with other potential outbreak and herd variables was evaluated. Significant clustering of positive herds was identified in the three years of the study in the same location (“high risk area”). Three spoligotypes (SB0339, SB0121 and SB1142) accounted for >70% of the outbreaks detected in the three years. VNTR subtyping revealed the presence of few but highly prevalent strains within the high risk area, suggesting maintained transmission in the area. The spatial autocorrelation found in the distribution of the estimated within-herd transmission coefficients in herds located within distances <14 km and the results of the spatial regression analysis, support the hypothesis of shared local factors affecting disease transmission in farms located at a close proximity.

## Introduction

Bovine tuberculosis (bTB), caused by members of *Mycobacterium tuberculosis* complex (mainly *M. bovis*, and to a lesser extent, *M. caprae*) is an important zoonotic disease with a global distribution that has major implications for both animal and human health [1]. Implementation of eradication programs, mainly through test-and-slaughter strategies, has led to a significant decrease of the prevalence and even to disease eradication in many developed countries [2], [3] However, success of eradication programs is not uniform, leading to the persistence of bTB in certain areas, usually associated with the presence of one or more known risk factors, such as wildlife reservoir, large herds, or extensive management that complicates animal testing [4]–[6].

The efficacy of eradication programs may be improved by using spatial statistics to detect clusters of persistent infection and consequently targeting control efforts to those high risk clusters [7], as shown previously for bovine tuberculosis in livestock [8], [9] and wildlife [10], [11]. Spatial analysis can be particularly powerful when combined with molecular typing techniques, such as spoligotyping [12], and MIRU-VNTR typing [13], which can provide critical information on the spatio-temporal patterns of pathogen transmission [14]. The use of those techniques has provided useful information of the distribution of *M. bovis*/*M. caprae* in a number of countries [15]–[18], demonstrated the transmission between livestock and wildlife [19], and provided useful information at a regional/local scale [11]. Strain-related differences in terms of transmissibility and/or virulence has been also suggested [20].

In Spain, a national bTB eradication program has been in place since 1987, resulting in a marked decrease of bTB herd prevalence (from 11.4% in 1986 to 1.31% in 2012), although progress has been heterogeneous. This program is nowadays based on yearly skin testing of every ≥6 weeks old animal, parallel use of the interferon-gamma (IFN-γ) assay as an ancillary diagnostic test in infected herds, slaughter of all reactors, control of livestock movement, slaughterhouse surveillance and monitoring, and control measures in wildlife reservoirs [21]. Despite the program's success, the disease is still present at levels of concern in some areas, such as the Autonomous Community of Madrid (ACM) [21], in which a special incidence area was recently declared [22], thus suggesting the presence of factors impairing the progress of the eradication campaigns.

In the present study, a spatial analysis was carried out using bTB data collected in the ACM during a three-year period (2010–2012) with the objective of identifying clusters of farms at high risk of disease. In addition, information on the molecular profiles of the *M. tuberculosis* complex strains isolated from cattle during the study was used to identify highly prevalent strains associated with persistent local transmission. Finally, differences in the within-herd transmission coefficients estimated at a herd level in infected herds were also assessed, as well as possible factors associated with high rates of disease transmission. Results found here will help to evaluate the progress of bTB control in ACM and to support the design and implementation of effective eradication programs in the regions.

## Materials and Methods

### 1. Study population

**Figure 1:**
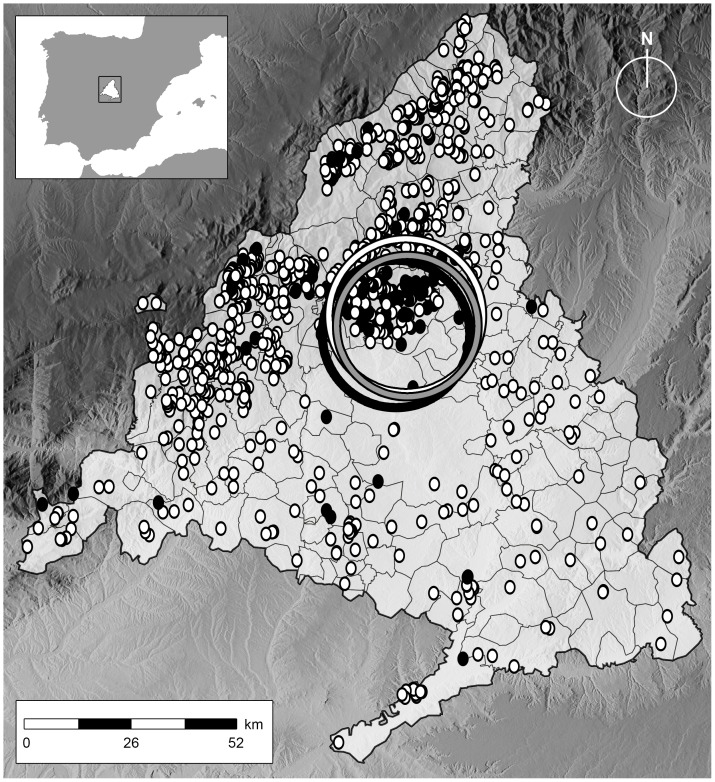
Location of cattle herds tested in the bTB eradication program during 2010–2012 in the ACM. Black dots indicate herds that were positive at some stage in 2010–2012, and white dots indicate herds consistently negative during the three years. Circles indicate clusters of high risk identified in 2010, 2011 and 2012.

The ACM, located in central Spain, covers an area of 8,022 km^2^, with a mean altitude of 650 m and a continental-Mediterranean climate. During the 2010–2012 study period, ACM was home to 1,496 cattle herds that were tested at least once during the 3 year study period in the frame of the eradication program. Only herds for which the spatial location (coordinates) was available to us (n = 1,387, 92.7%) were included in the study (Fig. 1). The predominant production type was beef (83.3% of the herds), followed by bullfighting (6.9%) and dairy (6.1%), whereas the remaining herds (<4%) were of mixed types. Information included in the study was exclusively derived from the work performed in the frame of the eradication programs, and thus no experimental research on animals was performed.

### 2. *In-vivo* tuberculosis diagnostic tests

All active cattle herds included in the program were subjected to at least one annual herd test using the single intradermal tuberculin (SIT) test according to the European and Spanish regulations [23], [24]. SIT tests were performed by field practitioners in all animals >6 weeks old by inoculation of 0.1 mg of the official bovine PPD (CZ Veterinaria, Porriño, Spain) in the left side of the neck. After 72 hours, animals with an increase of the skin fold thickness >2 mm were considered reactors (severe interpretation). In addition, animals >6 months old from positive herds (in which the infection was confirmed by detection of lesions, culture and/or epidemiological evidences) were also subjected to IFN-γ testing. Hence, the IFN-γ assay was used in those herds as an ancillary test to maximize the number of infected animals detected [23] as described elsewhere [25].

### 3. Post-mortem laboratory tests

All reactors were subjected to post-mortem analysis to confirm the infection: first, animals were screened to detect macroscopic lesions compatible with bTB in the slaughterhouse. Lesions (if present) or tissue samples (lung and retropharyngeal, bronchial and mediastinal lymph nodes) were collected from all reactors and processed in the laboratory for isolation of *M. tuberculosis* complex members [26]. Isolates were identified as *M. bovis*/*M. caprae* and characterized using spoligotyping [12]. Isolates from the most prevalent spoligotypes in areas of high prevalence were also typed using the Variable Number of Tandem Repeats (VNTR) method as described before [13] using four loci (ETR-A, ETR-B, QUB11a and 3232s) (S1 Table) previously described to be highly variable among *M. bovis* isolates from the Iberian peninsula [27], [28].

### 4. Data analysis

Information on the herd type (beef, dairy, bullfighting or other) and census, management (extensive/intensive), location and bTB test results (date, tests performed, number of animals tested, number of reactors at each test, number of *M. bovis*/*M. caprae* positive cultures, spoligotype/VNTR profiles found) during 2010–2012 were collected at the herd level from the official veterinary services and the MycoDB (http://www.mycodb.es) databases. Positive herds were those in which at least one reactor at the SIT test and/or the IFN-γ assay was disclosed in a herd-test during the study period. An outbreak was defined as the time period from first disclosure of a bTB reactor until the herd had recovered its official bTB-free (OTF) status. Proportions and quantitative outcomes obtained in different groups/clusters were compared using non-parametric tests (chi-square test, Mann-Whitney test, Kruskal-Wallis test). The evolution of the herd prevalence in the ACM was evaluated using the Cochrane-and-Armitage and Mantel tests for lineal trend. Cochrane–Orcutt procedure was applied to control serial correlation [29]. The discriminatory power (D) of spoligotyping and combined spoligo-VNTR typing, defined as the average probability that each technique will allocate two isolates randomly sampled from a population in two different groups, was calculated using the Hunter and Gaston equation [30]. For the analysis each different profile obtained from a given herd was counted once regardless the number of typed isolates sharing the same profile.

Clustering of positive herds/prevalent molecular profiles was assessed using the Bernoulli model of the spatial scan statistic. This model compares the observed number of bTB-positive herds within all possible circular spatial windows in the study area with the expected number of cases under the null hypothesis of random distribution of positive herds [31]. We limited candidate clusters to 50% of the total herds, and the region was scanned for areas at high and low risk for the disease. The test was implemented using the SaTScan software version 9.1.1 [32].

In addition, we estimated the transmission coefficient (β, the average number of animals infected from an infectious individual per unit of time) [33], [34] in positive herds with sufficient available information (date of first positive herd-test, size of the herds, and number of reactors at each diagnostic test). Estimates for β were calculated at the herd level as previously described with a slight modification: the sensitivity and specificity of the *in-vivo* diagnostic tests were assumed to range from 0.632 to 0.839 and 0.755 to 0.968, respectively, in agreement with previous findings in a similar epidemiological setting as the one studied here [35]. Values of β were computed after 1000 simulations using @RISK 5.5 (Palisade Co, Ithaca, NY, USA).

Incremental spatial autocorrelation (ISA) of the log-transformed β coefficients in the region was evaluated at increasing distances up to 22 km using Moran's I [36]. Before autocorrelation was assessed, spatially isolated herds (defined as herds separated from their nearest neighbor by more than three standard deviations of the nearest-neighbor distances for all herds) were removed. When Moran's I detected significant autocorrelation, the locations of any clusters were identified using the Getis-Ord G*_i_ statistic [37], which compared local β values (for each herd and its neighbors within various distance bands) with global values to identify areas with β values that were higher (“hot spots”) or lower than expected (“cold spots”). All autocorrelation tests were carried out using the Spatial Statistics Tools implemented in ArcGIS 10.1 SP1 (ESRI, Redlands, California, USA).

Possible association between the herd-level factors and the log-transformed β coefficient for each herd were identified using simple linear regression. Potentially significant (p<0.3) covariates were then included as main effects in a multiple regression model including significant (p<0.05) scientifically sound two-way interactions. The final model was selected by an all-possible-subset regression algorithm using the C_p_ criterion developed by Mallows [38]. Residuals of the final model were analyzed for spatial autocorrelation using Moran's test [36] to determine whether any spatial autocorrelation detected by the ISA above had been explained by the model for herd-level factors. For this analysis, the neighborhood for each herd was defined by the ISA distance band identified above with inverse-distance weighting between herds. When significant residual spatial autocorrelation was found, a conditional autoregressive (CAR) model [39] was fitted using the same covariates [40]. Regression analyses were carried out using R [41] and the spdep package for CAR models [42].

## Results

### 1. Distribution of bTB-positive farms

Herd-level prevalence (number of positive herds) was 5.5% (n = 72), 7.3% (n = 94), and 5.9% (n = 80) in 2010, 2011, and 2012 respectively, providing a non-significant trend for the evolution of the herd prevalence in the three-year period of study (p>0.05). Herd prevalence was not associated with production type (Chi-square test, p>0.05).

High risk clusters (p<0.05) were identified at approximately the same spatial location in each of the three years of study (Fig.1, Table 1). Up to 230 herds (16.6% of those included in the study) were included in a significant cluster at least once during the study period, 157 of which were consistently found in the area in which all three clusters overlapped (defined as “high risk area” from now on). A significantly higher proportion of bullfighting herds were found in the 230 herds included at least once in a significant cluster compared with the rest of the population (10.9% vs. 6.1%, Chi-square test, p = 0.012) although this difference became non-significant when the group was narrowed to include only the 157 herds consistently found in the high risk area (p = 0.12). No significant differences were observed in the herd size depending on the location (inside/outside high risk areas) (Mann-Whitney test, p = 0.3).

**Table 1:** Significant (p<0.05) clusters of bovine tuberculosis-positive herds found in each year during the period 2010–2012 in the Autonomous Community of Madrid.

Year	Radius (km)	Included herds	Cases Observed	Cases Expected	RR^a^	P-value
2010	14.6	217	37	11.9	5.35	<0.001
2011	13.4	154	56	11.3	10.8	<0.001
2012	14.0	144	43	9.15	9.00	<0.001

a Relative risk within the cluster.

### 2. Molecular characterization of bTB outbreaks

At least one positive *M. bovis* culture was obtained and characterized in 76.4% (55/72), 73.4% (69/94) and 72.5% (58/80) of the positive herds detected in 2010, 2011 and 2012 respectively. Overall *M. bovis* spoligotypes were available for 129 (72.9%) of the 179 herds that were positive at some stage during the study period; in 64 of them more than one (and up to seven) different spoligotype profiles were detected. A total of 30 spoligotypes were found, with three profiles accounting for >70% of the profiles found every year (SB0339: 36.0%; SB0121: 20.2%; and SB1142: 17.4%). The discriminatory index at the herd level was D = 0.81. A significant cluster of high prevalence (p = 0.027) was only found for SB1142, comprising most of the isolates located in the high risk area and in fact isolates belonging to this spoligotype were found significantly (Chi-square test, p = 0.001) more often within the high risk area than outside (with 85.7% of all the SB1142 spoligotypes identified in the ACM found within this area). The discriminatory index obtained including herds located in the high risk area was D = 0.75.

Two-hundred and eighteen isolates belonging to the three most prevalent spoligotypes in the high risk area (SB0339, n = 112; SB0121, n = 61 and SB1142, n = 45), recovered in the 2010–2012 period from 53 different herds, were subjected to VNTR-typing (1–18 VNTR-typed isolates per herd). Fifteen of these 53 herds were positive at least two years during the study period. The three spoligotypes were subdivided into 24 different combined spoligotype-VNTR patterns. Spoligotypes SB0339 and SB1142 were subdivided into eight and four VNTR subtypes respectively, although one of them accounted for the majority of the isolates (MV0006 for SB0339, present in 72/112 (64.3%) of the isolates, and MV0003, found in 41/45 (91.1%) SB1142 isolates) (S1 Table). In contrast, SB0121 was further subdivided into 12 VNTR types, of which MV0001 and MV0074, the most abundant, were observed in only 16 (26.2%) isolates each of the 61 SB0121 typed strains (S1 Table). No significant (p>0.05) clustering of any spoligotype-VNTR pattern was identified within the high risk area. At the herd level the discriminatory index for the combination of spoligotyping and VNTR in the high risk area was 0.83.

More than one spoligotype-VNTR profile was observed in 22 (41.5%) of the 53 herds in the high risk area. In 12 of the 15 recurrently positive herds (positive at least two years) in 2010–2012 more than one profile was found (OR compared with non-recurrently positive herds  = 11.2, 95% CI = 2.6–48).

### 3. Spatial distribution of the β coefficient

Estimated within-herd transmission coefficients, β, were available for 152 bTB outbreaks in 142 cattle herds during 2010–2012 in Madrid, of which 143 occurred in 133 cattle herds (114 beef, 13 dairy and 6 bullfighting) with a known spatial location. Sixty-one of these herds were located within the high risk area. The median β estimate was 4.42 (IQR = 2.62–6.29). No significant differences were found between locations of the outbreaks (within/outside the high risk area; Mann-Whitney test, p = 0.6) or production types (Kruskal-Wallis test, p = 0.25). In contrast, β was significantly higher (Mann-Whitney test, p<0.001) in herds in which the IFN-γ assay had been applied (n = 87, median β = 4.85) compared to those where only the SIT test was used (n = 46, median β = 2.6) and in those where *M. bovis* was isolated (n = 100, median β = 4.67) compared with those where it was not (n = 33, β = 2.58).

**Figure 2:**
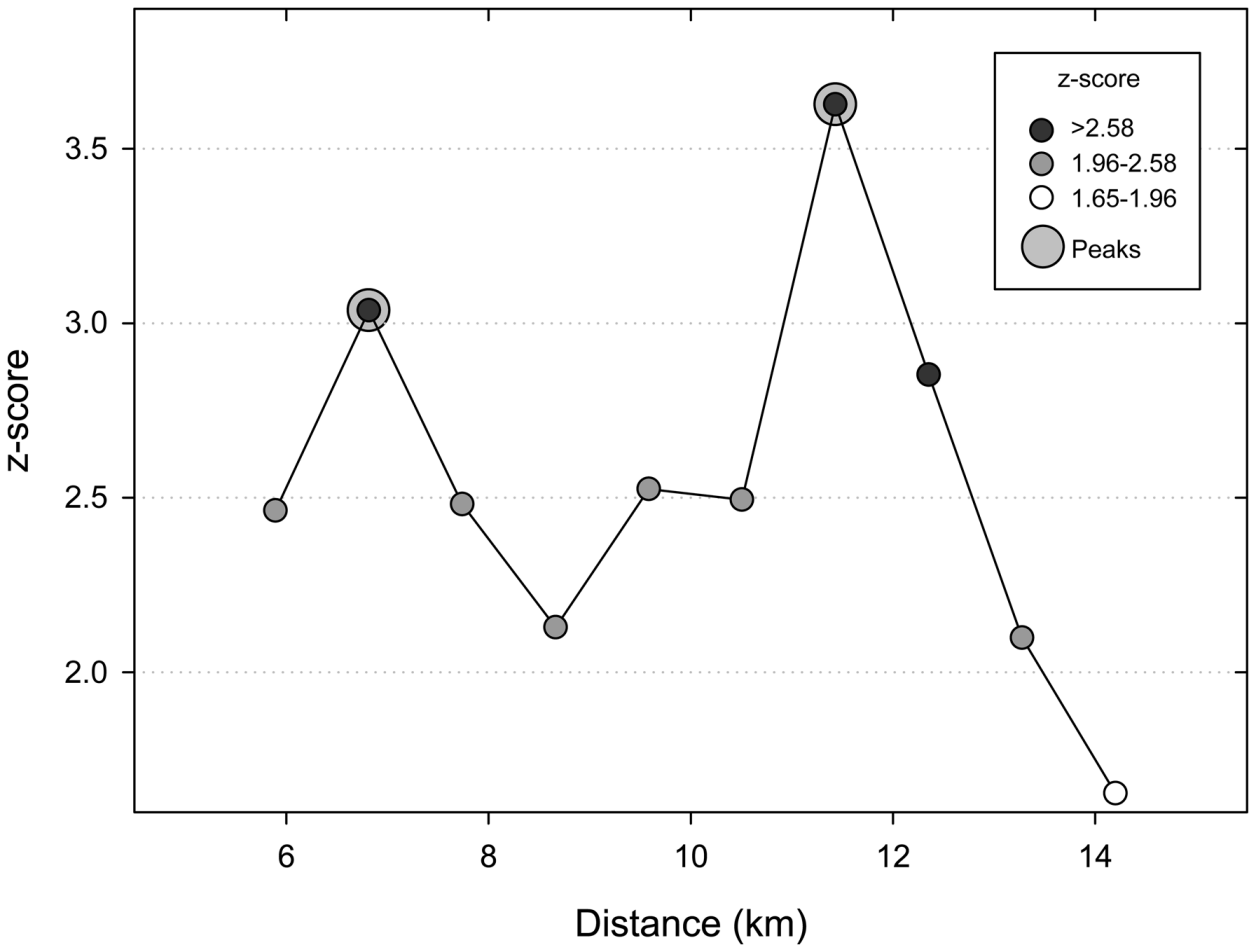
ISA of the log-transformed β coefficient of bTB –infected herds located in a high risk area (n = 60) in the ACM in 2010–2012.

**Figure 3:**
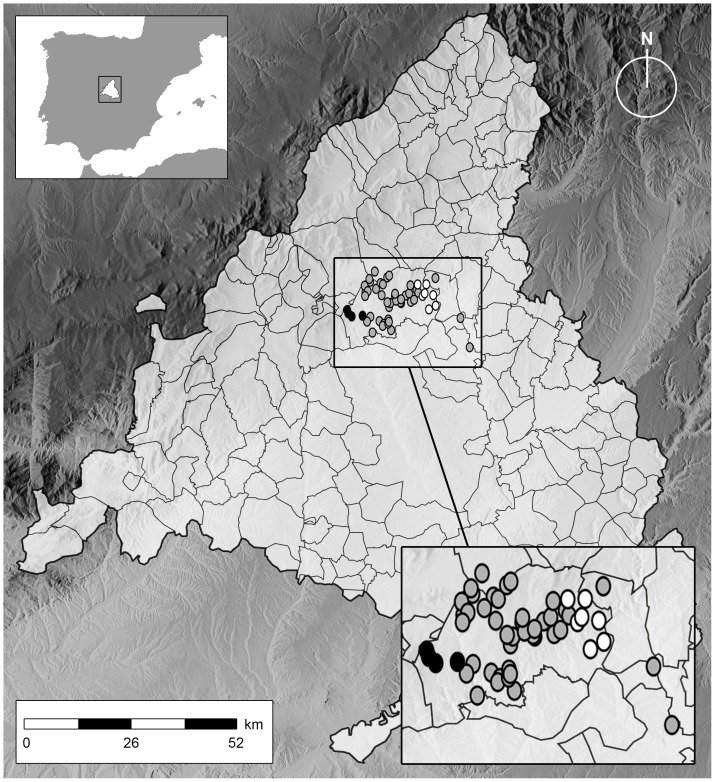
Cluster of herds showing significantly (p<0.05) low (black dots) or high (white dots) values for the log-transformed β coefficient as determined by Getis-Ord G*_i_ within a high risk area of the ACM.

The analysis of the log-transformed βs estimated in herds from the whole ACM (n = 133) suggested the absence of clustered transmission dynamics (Moran's I = 0.40, Z = 1.32, p = 0.19) once spatial outliers (n = 6) were removed. Still, incremental spatial autocorrelation analysis revealed that most clustering, although not significant (p = 0.09), occurred at 14 km. When the analysis was performed using only infected herds located in the high risk area (n = 60), incremental spatial correlation analysis of the log-transformed βs revealed the occurrence of highly significant positive autocorrelation (p = 0.0003–0.036) between 6 and 14 km distance with a peak at 12 km (Fig. 2). In the high risk area, Getis-Ord G*_i_ revealed the presence of hot and cold spots with unexpectedly high and low log-transformed β values (*p<*0.05) in the western and eastern parts of the area respectively, including 5 and 7 herds (Fig. 3).

### 4. Regression analysis

Bivariate linear regressions using data from all the ACM failed to identify any herd- or management-related variable associated with disease. However, there were six bTB-related variables as potentially (p<0.3) associated with the log-transformed β coefficient: number of reactors in the first herd test, number of positive herd-tests in the outbreak, use of IFN-γ, isolation of *M. bovis* during the outbreak, number of spoligotypes present and existence of previous outbreaks). The final multivariable model included only the number of reactors in the first herd-test, use of IFN-γ assay, isolation of *M. bovis* and the interaction between the latter two (Table 2). Moran's I test for spatial autocorrelation in residuals from this model did not detect any evidence of spatial correlation (p = 0.20).

**Table 2:** Point estimates, standard errors (S.E.) and 95% confidence intervals (95% CI) of the regression coefficients (β) and P-value of the variables remaining in the final models measuring the association between herd-level covariates and the log-transformed within-herd transmission coefficient for all infected herds located in the Autonomous Community of Madrid in 2010-2012 (n = 246) and infected herds located in a high risk area (n = 65).

Population studied	Variable	β	S.E.	P
All herds	reactors in first herd-test	0.047	0.008	<0.001
	use of IFN-γ	0.397	0.095	<0.001
	History of previous outbreaks	−0.211	0.109	0.05
Herds in high risk area	reactors in first herd-test	0.054	0.009	<0.001
	Isolation of *M. bovis*	0.574	0.203	0.006
	History of previous outbreaks	−0.255	0.133	0.06

Bivariate analysis of data from herds located in the high risk area identified a slightly different set of production (management type and categorized census) and outbreak-associated (number of reactors in the first herd test, use of IFN-γ, isolation of *M. bovis* during the outbreak and existence of previous outbreaks) variables as potentially associated with the log-transformed β coefficient. The final model included only number of positive reactors in the first herd test, isolation of *M. bovis* and existence of previous outbreaks. However, in this case Moran's I test for spatial autocorrelation in residuals from the model suggested the presence of spatial correlation (p = 0.06). Consequently, a CAR model was fitted, which resulted in controlling for spatial autocorrelation (λ = 6.69*10^−5^, p = 0.15) (Table 2).

## Discussion

Despite the efforts and funds invested on bTB control programs, disease eradication from the cattle population still remains a challenge in many developed countries. Even within a single country, the results of national eradication programs may be highly variable. This heterogeneity is often linked to the presence of a significant wildlife reservoir that maintains the infection [43], but may occur also in areas such as the ACM in which prevalence of infection in the potential wildlife reservoir species present, the wild boar, is considered low. Here, we analyzed the spatial variation in bTB within the ACM to describe risk patterns and determine the causes of departures from typical transmission levels within the region.

Data from a last three-year period (2010–2012) revealed a consistent cluster of high prevalence (Fig. 1). This is not surprising, given the considerable time required for regaining the OTF status in infected herds, with a median duration of 307 days in the period 2006–2012 in the same region [35]. The lack of additional clusters in other areas in which estimates of wild boar density are similar or higher than in the high risk area [44], [45] suggest that at least this is not the only factor playing a role in the maintenance of bTB in the region.

Given the limitations of bTB diagnostic techniques, isolation of the causative agent is desirable (and usually required) to confirm the infection at the herd level. In this study, we were able to confirm the infection in 75.6% of the positive herds, and at least one isolate was characterized using spoligotyping in 96.9% of them (approximately 73% of all detected outbreaks). Molecular typing data revealed the presence of three highly prevalent spoligotypes in the region, two of which (SB0121 and SB0339) are among the 15 most abundant profiles in a database of *M. bovis* isolates collected throughout all Spain in 1992–2007 [15]. Discriminatory power of spoligotyping in the region was lower than that described for the whole country (D = 0.81 vs. D = 0.87) [15]. In fact, finding that more than 70% of the profiles belonged to just three spoligotypes highlights the limitations of this technique for epidemiological purposes. This is particularly true when spoligotypes highly prevalent at the country level are found [46], as with no further information it is impossible to conclude if finding them is due to the recirculation of a limited number of highly prevalent strains or to a lack of discriminatory power of the technique.

For this reason isolates belonging to the three most prevalent spoligotypes in the high risk area were further subtyped using VNTR analysis. However, although the use of VNTR subtyping increased the discriminatory ability, a considerable genetic homogeneity was still found for SB0339 and SB1142 (with one spoligo-VNTR pattern accounting for 60–90% of the isolates) (S1 Table). The larger variability in isolates with a SB0121 spoligotype found here was in agreement with a previous study in which a panel of 115 SB0121 *M. bovis* isolates was further divided in 65 VNTR profiles [28]. Still, a more limited variability was observed here (D = 0.83 compared with D = 0.99), probably related with the increased probability of finding isolates epidemiologically related. At the national level SB0339 and SB1142 are found predominantly in the ACM (up to November 2013 52% and 78.2% of all typed isolates belonging to these profiles were coming from cattle located in this region according to the Spanish database of animal mycobacteriosis) [47]. Therefore, the limited genetic variability found in those spoligotypes may reflect the endemicity of a reduced number of spoligo-VNTR types widely distributed in the region. On the other hand, the higher heterogeneity found in isolates from the SB0121 spoligotype, the most abundant in the Iberian Peninsula [48], [15], could be a consequence of several different and unrelated introductions of *M. bovis* strains from other regions of the country. Thus, usefulness in outbreak investigation of VNTR to determine the potential relatedness of isolates with an epidemiological link would be highly dependent on the spoligotype of the isolates being typed. Still, the association between a higher number of spoligo-VNTR patterns found in a given herd and its positivity for at least two years in 2010–2012 suggests that recurrence of bTB in the herd in this three-year period may be related not only with persistence of infection but also to exogenous introductions of different *M. bovis* strains.

Within-herd transmission coefficient estimates found in the present study were in line with those obtained in the ACM [35] and elsewhere [49]–[51]. Horizontal transmission of bTB is considered to be low under normal circumstances, but certain management factors (such as herd size and density, or lack of hygienic measures in place) may contribute to increase the cattle-to-cattle transmission [52], [53]. In addition, the ancillary use of more sensitive diagnostic techniques, such as the IFN-γ detection assay, may induce an increase in the number of positive animals being detected, yielding an apparent increased transmission within a herd [35]. Therefore finding herd and outbreak-related variables potentially associated with the β estimate in a given herd was not an unexpected finding. Final models selected only variables associated with the outbreak (use of IFN-γ, number of reactors found in the first positive herd test and history of previous bTB outbreaks for the whole region and reactors in the first herd, history of previous outbreaks and isolation of *M. bovis* in the herd for the high risk area) reflecting the expected strong impact of increased number of reactors and of confirming the infection in the method used for β estimation. Although a prior history of disease in the herd has been associated previously with an increased risk of future outbreaks [54], here it was associated with a decreased estimate of β (both in the whole ACM and the high risk area) (Table 2). This would suggest that transmission was particularly low in herds experiencing repeated outbreaks compared with the rest of infected farms present in the region during the study period. Possible explanations for such finding include the presence of an increased rate of false-negative reactors in these farms (that could lead to an incorrectly lower estimate of within-herd transmission) or anergic infected cattle that would originate the different outbreaks. Interestingly the genetic profile of the isolate was not associated with the estimated transmission at the herd level; this could be due to a lack of strain-dependent differences in the transmissibility of a given strain or to the effect of the test-and-cull strategies that would hamper the expression of these differences at demonstrable levels [20].

The spatial pattern of the β coefficient across the region and particularly in the high risk area revealed a significant effect of the location of the herds in their estimates. A strong positive autocorrelation was found at low distances (<14 km) suggesting that the intensity of transmission within neighboring herds was similar. Such result may be due to a shared contact network (between-herd contacts in shared pastures, controlled and uncontrolled local cattle movements, exposure to infected wildlife or other as yet unidentified local source in herds located in the same vicinity). Neighboring contacts can be highly variable between and within regions [55], and no information was available here to further characterize the contact networks between farms in the ACM. However, the lack of autocorrelation in the residuals of the best model found for the whole region suggests that at the regional level variables included in the final model (use of IFN-γ assay, number of reactors in the first herd test and previous history of bovine tuberculosis) may account for the majority of the spatial variation of the β coefficient. At a local scale in the high risk area, evidences suggesting the existence of autocorrelation in the residuals of the best linear regression model led to the use of a CAR model, selected due to its best fit for situations with relatively local spatial autocorrelation [56], as those shown by the incremental spatial autocorrelation analysis here. The lack of autocorrelation in the residuals once the CAR model was fit supports the hypothesis of shared local factors affecting disease transmission in farms located at a close proximity (<14 km) within the high risk area.

In summary, bTB was unevenly distributed in the ACM, with a cluster of increased incidence being present in 2010–2012. Most cases (>70%) detected in the region were due to three spoligotypes (SB0339, SB0121 and SB1142). VNTR analysis of isolates from the high risk area belonging to these profiles revealed a high genetic homogeneity of isolates belonging to SB0339 and SB1142, thus suggesting a wide distribution of certain strains with a sustained transmission in the area. Within-herd transmission coefficient estimates were in agreement with those reported earlier for the same area, but the strong spatial dependence between the data, particularly in the high risk area, found at distances <14 km further supports the hypothesis of local transmission of strains between neighbor herds, or from a common source. These results will help to evaluate progress and to increase the effectiveness of bTB control programs in the region.

## Supporting Information

S1 Table VNTR subtypes of the three more prevalent spoligotypes recovered from the high risk area during the study period.
(DOCX)
