# Supplementary material for: Spatial Dynamics of Bovine Tuberculosis in the Autonomous Community of Madrid, Spain (2010–2012)
Source: PLoS One. 2014 Dec 23;9(12):e115632. doi: 10.1371/journal.pone.0115632 (PMC4275235; doi:10.1371/journal.pone.0115632)
Supplement: S1 Table — VNTR subtypes of the three more prevalent spoligotypes recovered from the high risk area during the study period. (DOCX) [file pone.0115632.s001.docx]

S1 Table. VNTR subtypes of the three more prevalent spoligotypes recovered from the high risk area during the study period.

| Spoligotype | VNTR subtypes | MIRU-VNTR allelic profile | | | | N |
| --- | --- | --- | --- | --- | --- | --- |
|  |  | ETR-A | ETR-B | QUB11a | 3232s |  |
| SB0121 (n=61) | MV0001 | 6 | 4 | 10 | 7 | 16 |
|  | MV0002 | 5 | 4 | 10 | 8 | 3 |
|  | MV0003 | 4 | 4 | 10 | 8 | 1 |
|  | MV0005 | 5 | 2 | 7 | 6 | 12 |
|  | MV0009 | 5 | 3 | 10 | 7 | 1 |
|  | MV0016 | 5 | 4 | 5 | 5 | 1 |
|  | MV0026 | 5 | 3 | 10 | 8 | 1 |
|  | MV0070 | 7 | 4 | 9 | 9 | 2 |
|  | MV0074 | 6 | 4 | 9 | 7 | 16 |
|  | MV0111 | 5 | 2 | 7 | 9 | 2 |
|  | MV0186 | 5 | 4 | 9 | 8 | 1 |
|  | MV0187 | 6 | 4 | >12 | 13 | 5 |
| SB0339 (n=112) | MV0002 | 5 | 4 | 10 | 8 | 8 |
|  | MV0006 | 5 | 4 | 10 | 7 | 72 |
|  | MV0012 | 5 | 4 | 7 | 7 | 1 |
|  | MV0028 | 5 | 5 | 10 | 8 | 1 |
|  | MV0061 | 4 | 4 | 10 | 7 | 26 |
|  | MV0087 | 2 | 4 | 10 | 7 | 1 |
|  | MV0112 | 5 | 2 | 10 | 7 | 2 |
|  | MV0186 | 5 | 4 | 9 | 8 | 1 |
| SB1142 (n=45) | MV0003 | 4 | 4 | 10 | 8 | 41 |
|  | MV0006 | 5 | 4 | 10 | 7 | 1 |
|  | MV0023 | 4 | 4 | 10 | 9 | 2 |
|  | MV0061 | 4 | 4 | 10 | 7 | 1 |
